# Coaching tumor-infiltrating CD8^+^ T cells to eat right

**DOI:** 10.18632/oncoscience.390

**Published:** 2018-01-22

**Authors:** Ral Kurupati, Hildegund CJ Ertl

**Affiliations:** Wistar Institute, Philadelphia, PA, 19104, USA

At least in experimental animals the efficacy of active immunotherapy of cancer can be increased by metabolic reprogramming of tumor antigen (TA) specific CD8^+^ T cells towards fatty acid (FA) catabolism and away from glycolysis [1]. In a nutshell, TA-specific CD8^+^ T cells or T cells with a chimeric antigen receptor (CAR) directed to a surface-expressed TA are hampered in their efforts to combat cancers by rapid loss of functions once they enter the tumor microenvironment (TME). This exhaustion, accompanied by increased cell surface expression of check point inhibitors was originally attributed to chronic TA-driven stimulation of CD8^+^ tumor infiltrating lymphocytes (TILs)[2]. Exhaustion of bystander CD8^+^ TILs, directed to antigens not present within the TME, contradicts this notion but rather points towards a different mechanism.

T cells during activation in the periphery switch to glycolysis to meet their demand for energy and biomass production. Upon entering solid tumors they encounter an environment that is metabolically challenging and provides opposing cues. Lack of oxygen due to insufficient vessel formation in rapidly growing tumors increases signaling through the hypoxia-induced factor (HIF)-1a pathway, which promotes glycolysis. Glycolysis obviously requires consumption of glucose, which is commonly scarce in solid tumors, as it is also the cancer cells' preferred fuel. As a result, T cells conditioned towards glycolysis may starve within the TME. The interstitial fluid of solid tumors is commonly rich in fatty acids (FAs), which activate signaling through the peroxisome proliferator-activated receptor (PPAR)-α, a transcription factor that augments lipid metabolism. Indeed CD8^+^ T cells that survive the harsh TME and are still present in advanced tumors have managed to switch from glucose to FA catabolism [1]. This metabolic switch could be thorny; it requires oxygen, which can also be limiting in solid tumors. But, tumors are not uniformly hypoxic. T cell migrate within tumors and will likely encounter areas of normoxia that can support FA beta oxidation (FAO), which not only provides energy through oxidative phosphorylation and intermediates for biomass synthesis but also initiates production of ketone bodies that can serve as nutrients in areas of hypoxia [3].

The loss of activated CD8^+^ T cells within the TME during tumor progression suggests that many T cells are unable to make this crucial metabolic switch. Like in any war it helps to train the troops before sending them into battle. CD8^+^ T cells taught to gain energy through FAs rather than glucose before they enter the TME fare better that those that are untutored; their functions remain preserved and they are more effective at delaying tumor progression [1].

There are many ways to change a cell's metabolism. Knockdown of HIF-1α or other key factors of the glycolysis pathway reduces a cell's reliance on glucose. The same effect could be achieved by reinforcing PD-1 signaling, which through blockade of the Akt/mTor pathways decreases glycolysis and increases lipid catabolism. FAO can be enhanced directly through strengthening PPAR-α signaling or genetically increasing the activity of rate-limiting steps of FAO, such as Carnitine Palmitoyltransferase (CPT 1), an enzyme that is essential to transfer long-chained FAs into mitochondria. Production of ketone bodies, which may provide crucial nutrients in hypoglycemic and hypoxic solid tumors, could be intensified by knock-in of 3-Hydroxy-3-Methylglutaryl-CoA Synthase (HMGCS) 2, the rate-limiting enzyme of ketogenesis.

Our efforts thus far have focused in fenofibrate [1], a PPAR-α agonist approved for treatment of humans with high cholesterol. TA-specific CD8^+^ T cells stimulated in mice treated with fenofibrate are more efficacious at reducing tumor burden than those activated without the drug. Fenofibrate upon entering mitochondria reversibly inhibits complex I of the electron transfer chain[7]. In serum and within the cytoplasm it is desterized into fenofibric acid, which retains PPAR-α activity but fails to cross mitochondrial membrane. As our recent results show, fenofibric acid outperforms fenofibrate for in vitro reprogramming of TA-specific CD8^+^ T cells and should thus be explored further (unpublished)*.

Recent years have shown tremendous progress in active immunotherapy of cancers through TA-specific vaccines[5], adoptive T cell transfer[6] or check point inhibitors[7]. The effectiveness of such treatments could be enhanced by pre-adjusting the metabolism of TA-specific CD8^+^ T cells to better prepare them for the hostile conditions of a TME. Treatment would need to be targeted to the unique metabolic conditions with different types of cancer.
